# Pathway-Dependent Coordination Networks: Crystals versus Films

**DOI:** 10.1021/jacs.1c08087

**Published:** 2021-10-07

**Authors:** Naveen Malik, Vivek Singh, Linda J. W. Shimon, Lothar Houben, Michal Lahav, Milko E. van der Boom

**Affiliations:** † Department of Molecular Chemistry and Materials Science, The Weizmann Institute of Science, 7610001 Rehovot, Israel; ‡ Department of Chemical Research Support, The Weizmann Institute of Science, 7610001 Rehovot, Israel

## Abstract

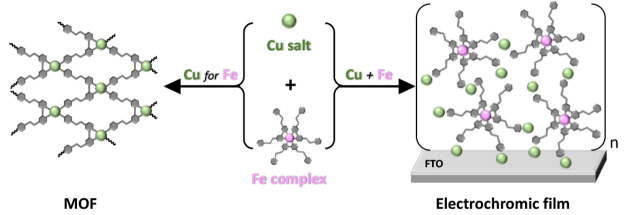

We demonstrate the formation of both metallo-organic crystals *and* nanoscale films that have entirely different compositions and structures despite using the same set of starting materials. This difference is the result of an unexpected cation exchange process. The reaction of an iron polypyridyl complex with a copper salt by diffusion of one solution into another resulted in iron-to-copper exchange, concurrent ligand rearrangement, and the formation of metal–organic frameworks (MOFs). This observation shows that polypyridyl complexes can be used as expendable precursors for the growth of MOFs. In contrast, alternative depositions of the iron polypyridyl complex with a copper salt by automated spin coating on conductive metal oxides resulted in the formation of electrochromic coatings, and the structure and redox properties of the iron complex were retained. The possibility to form such different networks from the same set of molecular building blocks by “in solution” versus “on surface” coordination chemistry broadens the synthetic space to design functional materials.

Coordination chemistry has been used to control the shape, size, and topology of supramolecular structures and to limit the possibilities to produce mixtures of multiple sets of structures.^1−3^ Early examples of self-assembled architectures are compounds made from cryptands and crown ethers and were studied by Pederson, Lehn, and Cram in the 1960s.^4−6^ In the following years, highly complex helicates were introduced and are composed of oligobipyridine strands coordinated to copper cations.^7−10^ The coordination chemistry of carboxylic acids and late transition metals has been extensively used for the formation of metal–organic frameworks (MOFs).^11−15^ This strong metal–ligand interaction resulted in highly robust and porous materials. Metal–pyridine coordination chemistry has also been used for the generation of self-assembled structures in solution and on surfaces.^16−35^ Although the interaction between metals and pyridine is weaker than with carboxylic acids, stable and functional materials can be isolated. Examples include cages,^18−22^ MOFs,^23−25^ and thin films.^26−35^ The control of material properties by external stimuli (e.g., light, voltage) has resulted in diverse functionalities, including memory elements,^16,17^ biomedical applications,^21^ and electrochromism.^26−35^

Structures and functionalities of supramolecular assemblies formed in solution or on surfaces are difficult to predict. Assemblies formed from the same starting materials can have the same or different molecular arrangements and function.^36−44^ Interfacial chemistry has been used to generate assemblies that cannot be formed in solution otherwise.^45^ Monolayer chemistry can be applied to control the chirality and morphology of crystals and network interpenetration of MOFs.^40,46,47^ In general, the development of defined supramolecular structures with desirable properties occurs with retention of the structural integrity of the molecular building blocks, but the assemblies can have different molecular arrangements and appearances.^36−47^ Synthetic routes that involve changes in the molecular structures prior to assembly of the components are rare,^43,44^ and examples of the pathway dependence of such processes are unknown to the best of our knowledge.

Here we show the formation of two assemblies having strikingly different molecular compositions, although the same starting materials were used (Schemes 1 and 2). Reacting a structurally well-defined iron polypyridyl complex with copper nitrate by diffusion of one solution into another resulted in exchange of the metal cations followed by the formation of MOFs. The initial disassembly of the iron complex is followed by the formation of a coordination polymer consisting of the polypyridyl ligand and copper cations. In contrast, alternative spin coating of the same iron polypyridyl complex with copper nitrate on fluorine-doped tin oxide (FTO) resulted in electrochromic coatings. Their electrochromic activity originates from the iron polypyridyl complex. The free pyridine moieties of the polypyridyl ligands are coordinated to the copper cations, forming a dense network of iron complexes. This network stabilizes the iron complexes, as cation exchange was not observed, even after prolonged exposure to a solution containing an excess of copper salt.

**Scheme 1:**
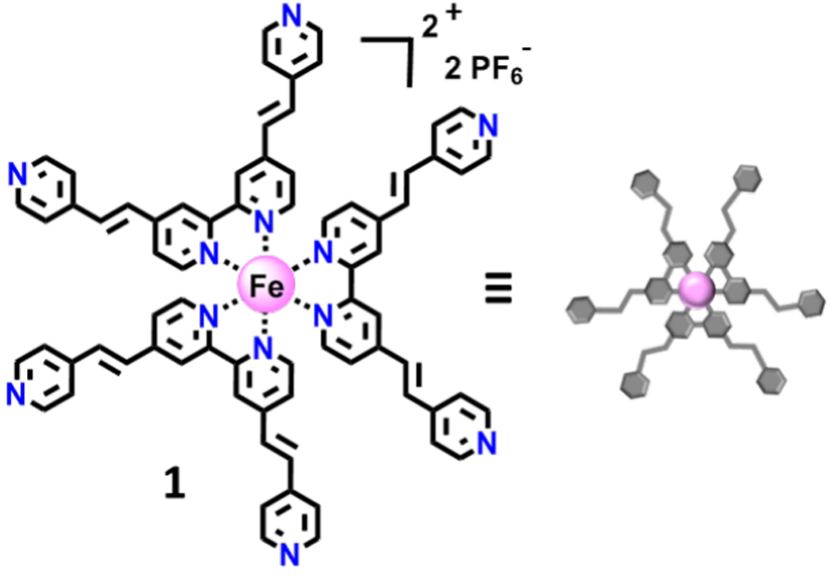
Iron Polypyridyl Complex **1**

**Scheme 2:**
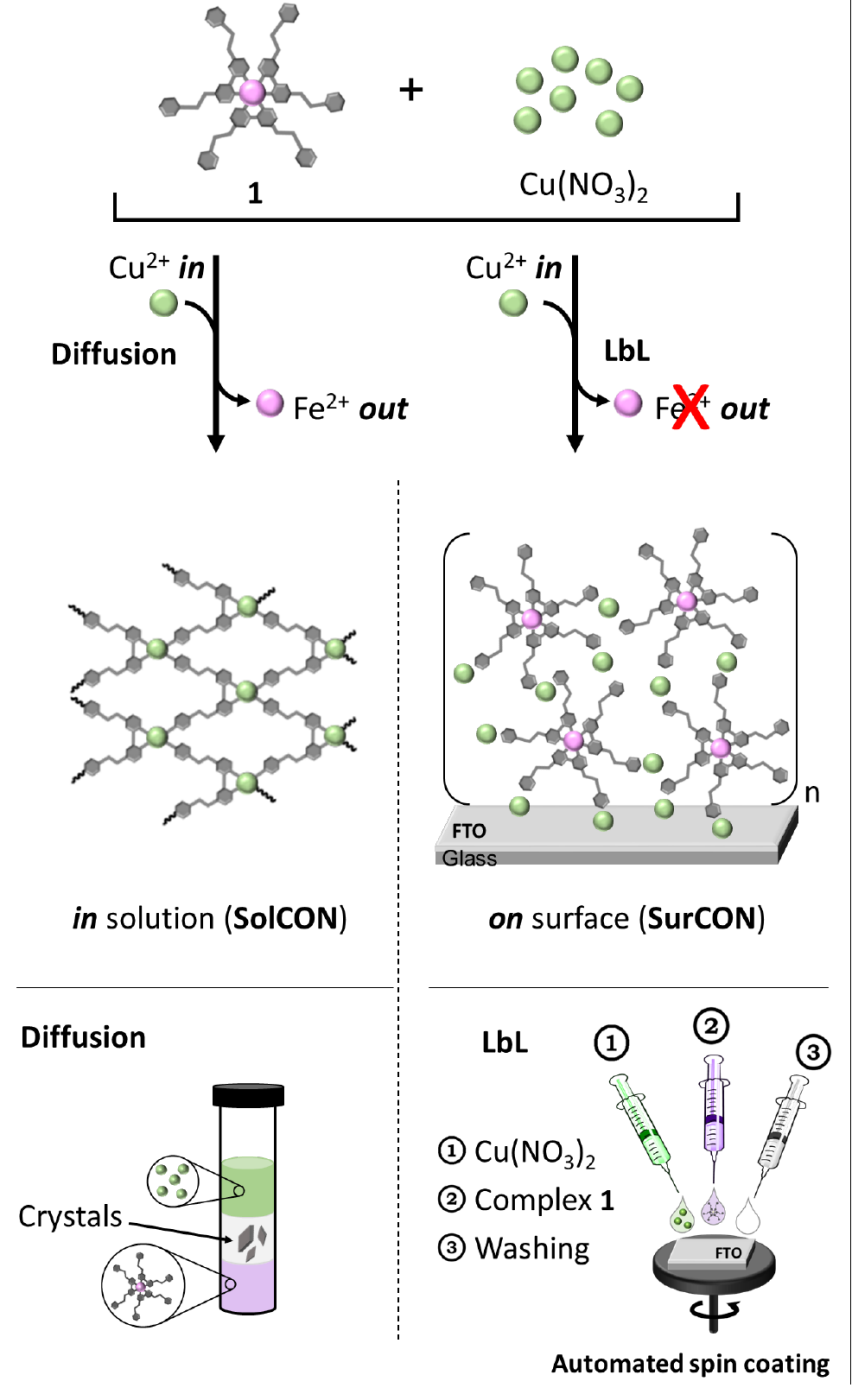
Formation of Divergent Coordination Networks in Solution (**SolCON**) versus on a Surface (**SurCON**) Water molecules and anions have been omitted for clarity. The diffusion experiment shown is for **SolCON-A**. For **SolCON-B**, the solutions with iron complex **1** and the copper salt are the top and bottom layers, respectively. LbL = layer-by-layer.

Crystals were obtained by slow diffusion of solutions into one another at room temperature. We used a thin tube (ø = 5 mm) containing three layers, with the top and bottom layers consisting of solutions of complex **1** or Cu(NO_3_)_2_ and the layer in the center being a cosolvent. During the reaction, the color of the solution changed from purple to colorless. These coordination organic networks (**SolCONs**) were isolated after 20 days by centrifugation and washed with acetonitrile (ACN) and ethanol. Two different solvent combinations were used and resulted in the same crystallographic structures and morphologies, but with slightly different dimensions.

**SolCON-A** was formed by the reaction of complex **1** (CH_2_Cl_2_/MeOH, 1:1 v/v) with Cu(NO_3_)_2_·3H_2_O in ACN in a molar ratio of 1:2. CH_2_Cl_2_/MeOH/ACN (0.5:0.5:1 v/v/v) was used as a cosolvent in the center. **SolCON-B** was obtained by using ACN as the solvent for complex **1** and *N*,*N*-dimethylformamide (DMF) as the solvent for Cu(NO_3_)_2_·3H_2_O. ACN/DMF (1:1 v/v) was used as a cosolvent in the center (Figure 1). Scanning electron microscopy (SEM) analysis revealed the formation of crystals that have the appearance of a parallelepiped (Figure 1, Chart 1). Although these crystals were uniformly shaped, their dimensions varied. For **SolCON-A** the size distribution was 2.1 ± 0.9 μm (∼50 crystals), and for **SolCON-B** sizes were between 1 and 4 μm (∼50 crystals). In addition, larger crystals of 38–70 μm (∼10 crystals) were also observed for **SolCON-B** (Figure S1). The different solvent combinations did not affect the overall crystal morphology, as is sometimes observed.^48^

**Figure 1:**
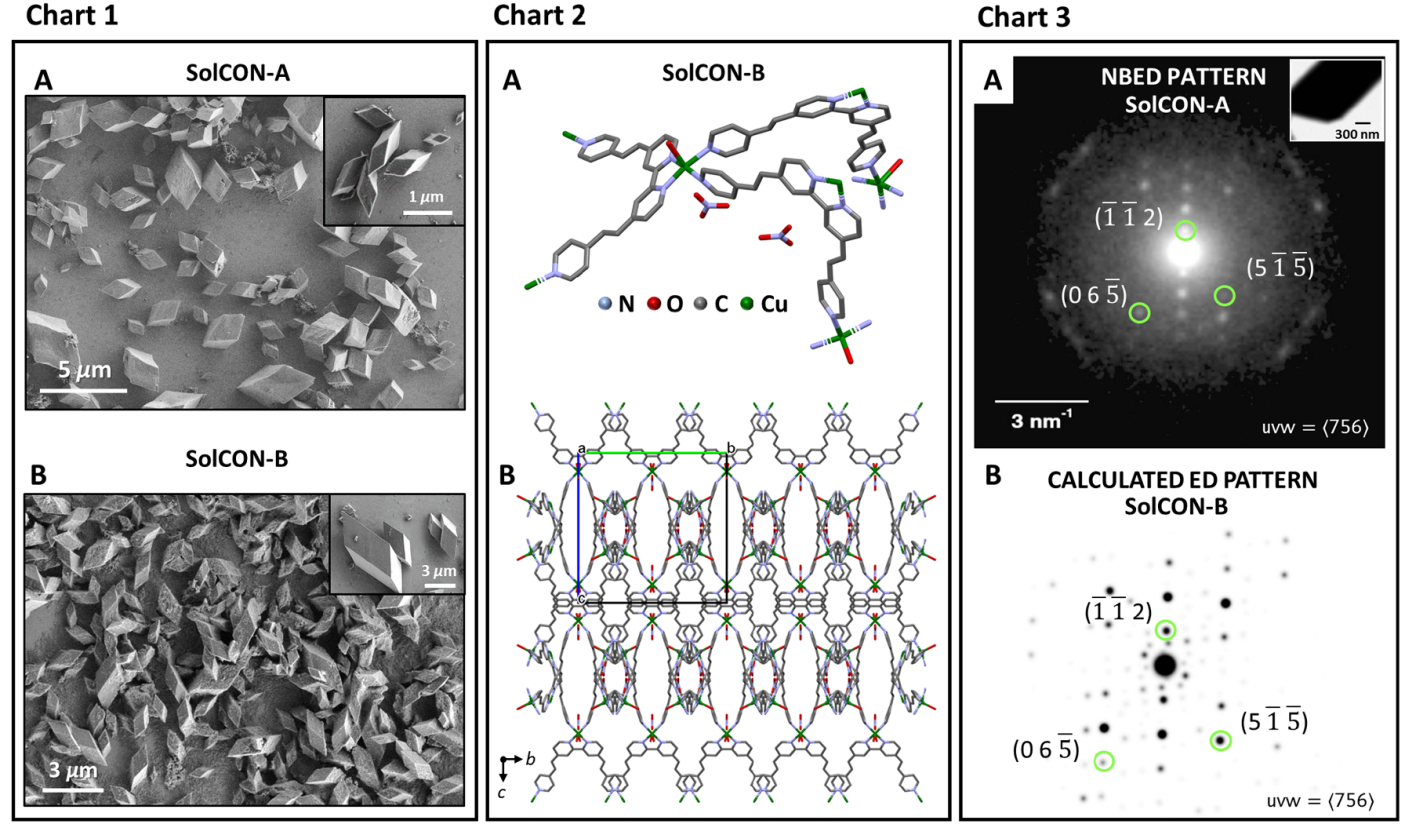
Characterization of **SolCON-A** and **SolCON-B**. Chart 1: (A, B) Scanning electron microscopy (SEM) images. Insets: Zoom-in. Chart 2: (A) X-ray analysis of **SolCON-B** (CCDC 2095187) showing the coordination environment of the polypyridyl ligand with the copper cations. Hydrogen atoms have been omitted for clarity. (B) Structural packing of **SolCON-B**. The view along the crystallographic *a* axis is shown. Chart 3: (A) Experimental nanobeam electron diffraction (NBED) pattern of **SolCON-A**. (B) Electron diffraction (ED) pattern calculated from the single-crystal X-ray structure of **SolCON-B**. The green circles indicate specific Miller planes of the matching zone-axis pattern.

Single-crystal X-ray analysis of **SolCON-B** showed the formation of a MOF based on copper cations and the ligand of complex **1** (Figure 1, Chart 2). The formation of the framework involved ligand transfer from complex **1** to the copper salt. The three-dimensional framework is formed by mono- and bidentate binding of copper centers to the pyridine moieties of the ligand. The ligands are coordinated in square-pyramidal fashion around the copper centers. The Irving–Williams series indicates that the relative stability of the copper complexes is expected to be larger than that of the iron complex **1**.^49^ Although structurally different, both coordination complexes have six metal–pyridine bonds. The N_pyr_–Cu^2+^ bonds are known to be stronger than N_pyr_−Fe^2+^.^50,51^ Two nitrate counteranions are present in the asymmetric unit, and hydrogen bonding is observed between the oxygen atom of NO_3_^–^ and hydrogen atoms of the polypyridyl ligand. The nanobeam electron diffraction (NBED) patterns of **SolCON-A** consist of sharp spots that match with the corresponding zone-axis patterns calculated from the refined structure of **SolCON-B**, demonstrating that these MOFs have very similar crystallographic structures (Figure 1, Chart 3, and Figure S2). The iron center of complex **1** is coordinately saturated, and therefore, it is highly likely that the metal cation exchange involves ligand dissociation prior to the formation of the MOFs.^52^ The vinylpyridyl moieties of complex **1** are not essential for the cation exchange, as shown by the reaction of [Fe(bpy)_3_](PF_6_)_2_ (lacking the vinylpyridyl moieties) with Cu(NO_3_)_2_ (40 equiv) in ACN. We observed the disappearance of the typical red color associated with this iron complex within 60 h.^53^

To demonstrate the differences between bulk crystallization and on-surface chemistry, a thin film (**SurCON**) was prepared by layer-by-layer (LbL) deposition of solutions containing complex **1** and Cu(NO_3_)_2_ (Figure 2). With this approach, complex **1** retains its structure and electrochromic properties. **SurCON** was assembled on FTO on glass (2 cm × 2 cm) using automated spin coating and solutions of Cu(NO_3_)_2_·3H_2_O (4.0 mM in ACN) and complex **1** (0.6 mM in CH_2_Cl_2_/MeOH, 1:1 v/v). This deposition sequence was repeated 18 times. The **SurCON** was coated with a thin layer of platinum and milled using a focused ion beam (FIB) microscope (Figure 2, Chart 1). The transmission electron microcopy (TEM) image of a cross section of **SurCON** shows a homogeneous film having a thickness of ∼178 nm. Energy-dispersive X-ray spectroscopy (EDS) mapping clearly indicates the uniform distribution of both the iron and copper cations.

**Figure 2:**
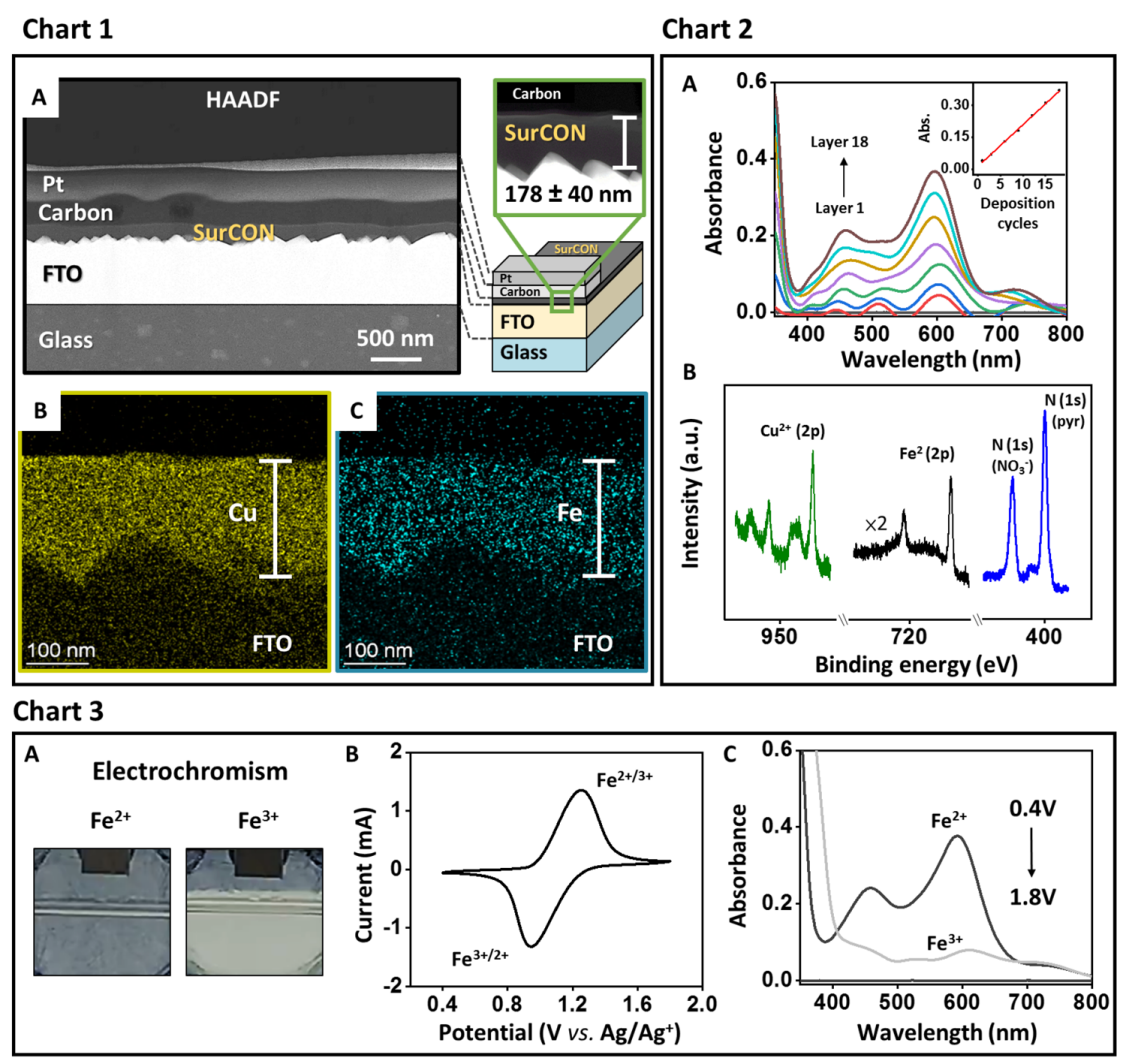
Characterization and electrochromic properties of **SurCON**. Chart 1: (A) High-angle annular dark-field scanning transmission electron microscopy (HAADF-STEM) image showing a cross section of a **SurCON** sample. (B, C) EDS elemental map showing the distribution of iron and copper metals. Scale bar: 100 nm. Chart 2: (A) Ex situ absorption spectra recorded during the formation of the film. FTO/glass was used for the baseline (black). Inset: Absorbance of the metal-to-ligand charge transfer (MLCT) band (λ_max_ = 596 nm) vs the number of deposition cycles. (B) X-ray photoelectron spectroscopy (XPS) spectra. Chart 3: (A) Photographs of the colored (0.4 V, Fe^2+^) and bleached (1.8 V, Fe^3+^) states using an electrolyte solution of 0.1 M TBAPF_6_ in ACN. Additional details are shown in Figure S3. (B) Cyclic voltammograms (CVs) recorded at a scan rate of 100 mV/s. (C) Absorption spectra showing the reduced (gray) and oxidized (light gray) states. FTO/glass was used for the baseline (black).

UV–vis spectra recorded for different numbers of deposition cycles showed the broad metal-to-ligand charge transfer (MLCT) bands related to complex **1** at λ_max1_ ≈ 458 nm and λ_max2_ ≈ 596 nm (Figure 2, Chart 2A). An intense π–π* transition band of the ligand was also present at λ_max_ ≈ 333 nm. Plotting the absorption intensity (λ_max_ ≈ 596 nm) versus the number of deposition cycles indicated linear growth with retention of complex **1**.

X-ray photoelectron spectroscopy (XPS) data for **SurCON** confirmed the presence of iron complex **1** and copper cations as cross-linkers (Figure 2, Chart 2B). Two characteristic bands for Fe^2+^ are present at 708 eV (2p_3/2_) and 720 eV (2p_1/2_).^34,54^ The N_pyr_/Fe ratio of 11.9 is in excellent agreement with the expected ratio for complex **1** (N_pyr_/Fe = 12). The bands for Cu^2+^ are observed at 935 eV (2p_3/2_) and 955 eV (2p_1/2_) along with the satellite bands at 941–945 and 962–965 eV.^54^ The observed Cu/Fe ratio (∼2.7) indicates the formation of a fully formed network (Cu/Fe = 3) in which the copper centers are bound by two pyridine groups.

Electrochemical measurements unambiguously confirmed the presence of the electrochromic complex **1** (Figure 2, Chart 3). Cyclic voltammograms (CVs) showed reversible one-electron redox processes as expected for the Fe^2+/3+^ couple with a half-wave potential (*E*_1/2_) of 1.1 V and a peak-to-peak separation of 310 mV at a scan rate of 100 mV/s. The color of the **SurCON** changed from gray (at 0.4 V) to colorless (at 1.8 V) upon oxidation of Fe^2+^ to Fe^3+^. This reversible process could be monitored using spectroelectrochemical (SEC) measurements (Figure S3). The changes in the oxidation states were accompanied by variations in the absorption intensities of the MLCT bands. The time required to reach 90% of the maximum transmittance (Δ*T* ∼ 40%) was ∼2.1 s. The switching stability was indicated by 250 redox cycles with >80% retention of the initial Δ*T*. The coloration efficiency (CE) was 148 cm^2^/C. **SurCON** is densely packed, as indicated by the molecular density of ∼1.1 × 10^16^ molecules/cm^2^ for a charge density (*Q*) of 1.77 mC/cm^2^. Exponential and linear dependences of the anodic and cathodic peak currents on the scan rate and square root of the scan rate, respectively, were observed, indicating a redox process controlled by diffusion. The calculated diffusion coefficients (*D*_f_) ≈ 3.37 × 10^–9^ cm^2^·s^–1^ (oxidation) and ∼3.64 × 10^–9^ cm^2^·s^–1^ (reduction) are similar and derived from the Randles–Sevcik equation. **SurCON** is remarkably stable, as no cation exchange was observable by UV–vis spectroscopy and electrochemical measurements. Immersion of **SurCON** in a solution containing Cu(NO_3_)_2_·3H_2_O (4.0 mM in ACN) for 3 days did not result in ligand transfer (Figure S4). Clearly, the formation of a network containing both the copper salt and complex **1** enhances its stability.

In conclusion, the reactions demonstrated here are two examples of coordination-based polymerization processes: (i) metal–ligand exchange followed by crystallization versus (ii) on-surface deposition. The composition of the assemblies is controlled by the applied method. We have shown that iron polypyridyl complexes can be used as sacrificial precursors for the formation of MOFs by slow diffusion of solutions. The on-surface polymerization is much faster, which prevents the metal–ligand exchange.

Fast mixing of solutions of iron complex **1** and Cu(NO_3_)_2_·3H_2_O resulted in a network without metal cation exchange, as shown by XPS, SEM, and EDS measurements (Figure S5). These observations suggest the formation of a kinetic product, whereas a thermodynamically favorable product is obtained by slow diffusion of the solvents. Reacting a palladium salt (instead of a copper salt) with the iron polypyridyl complex in solution (by diffusion or fast mixing; Figure S6) did not result in metal–ligand exchange.^55^

On-surface polymerization was observed by us with palladium salts for the formation of electrochromic coatings without metal cation exchange.^16,17,32,34,35,56^ Forming such coatings with copper rather than palladium salts is advantageous because of the lower toxicity and cost. The use of the same complex **1** resulted in similar properties (i.e., coloration efficiencies, maximum transmittance, and switching times) regardless of the applied salts.^32,34,35,56^ Others have reported the formation of related coordination structures based on terpyridine iron complexes and copper salts in solution and on surfaces.^29,36,37^ No exchange of the iron and copper cations has been reported. The previous finding with palladium chemistry and the above-mentioned reports^36,37^ by Constable, Housecroft, Gupta, Mondal, and Zharnikov highlight that our example of divergent coordination chemistry is rare and can offer new opportunities in the molecular engineering of functional materials.

## Supporting Information Available

Experimental details, materials, and methods; crystal data and structure refinement for **SolCON-B**; NBED of **SolCON-A**; spectroelectrochemical and electrochemical characterization of **SurCON**; characterization of the network formed from PdCl_2_(PhCN)_2_ and iron complex **1** (PDF)

## Accession Codes

CCDC 2095187 contains the supplementary crystallographic data for this paper. These data can be obtained free of charge via www.ccdc.cam.ac.uk/data_request/cif, or by emailing data_request@ccdc.cam.ac.uk, or by contacting The Cambridge Crystallographic Data Centre, 12 Union Road, Cambridge CB2 1EZ, U.K.; fax: +44 1223 336033.
