# Supplementary material for: Pathway-Dependent Coordination Networks: Crystals versus Films
Source: J Am Chem Soc. 2021 Oct 7;143(41):16913–8. doi: 10.1021/jacs.1c08087 (PMC8532112; doi:10.1021/jacs.1c08087)
Supplement: Supplementary file 1 — ja1c08087_si_001.pdf [file ja1c08087_si_001.pdf]

Supporting Information

Pathway-Dependent Coordination Networks: Crystals *versus* Films

Naveen Malik,[a] Vivek Singh,[a] Linda J. W. Shimon,[b] Lothar Houben,[b] Michal Lahav,*[a] and Milko E. van der Boom*[a]

[a]Department of Molecular Chemistry and Materials Science, and [b]Department of Chemical Research Support, Weizmann Institute of Science, 7610001 Rehovot, Israel. Email: michal.lahav@weizmann.ac.il; milko.vanderboom@weizmann.ac.il

EXPERIMENTAL SECTION

Materials and Methods

Solvents (AR-grade) were purchased from Bio-Lab (Jerusalem), Frutarom (Haifa, Israel), or Mallinckrodt Baker (Phillipsburg, NJ). Reagents were used without further purification. $Cu(NO_3)_2{\cdot}3H_2O$ (>98.0%) and tetrabutylammonium hexafluorophosphate ($TBAPF_6$) were purchased from Fluka Chemia and Sigma-Aldrich, respectively. Complex **1** was prepared according to reported procedure.[S1] Glass pressure tubes (Ace Glass, Inc., pressure tubes #15 with plunger valve, PTFE Bushing and FETFE® O-Ring, volume 50 mL) were cleaned by dipping in alkaline bath for 12 h. Subsequently, they were washed with deionized water and dried in an oven for 12 h at 130°C. Fluorine-doped tin oxide-(FTO)-coated glass substrates (2 cm × 2 cm, Rs = 10 Ω/□)[S2, S3] were purchased from Xinyan Technology, Ltd. (Hong Kong, China). FTO-coated glass substrates were cleaned by sonication in ethanol for 10 min, dried under a stream of N_2, and subsequently cleaned for 20 min with UV and ozone in a UVOCS cleaning system (Montgomery, PA). The substrates were then rinsed with tetrahydrofuran (THF), dried under a stream of N_2, and oven-dried at 130 °C for 2 h. A Laurell automatic spin-coater, model WS-65MZ-8NPPB, was used for the formation of the **SurCONs**.

Formation of SolCON-A. A Wilmad® screw-cap NMR tube (⌀ = 5 mm and length = 17.8 cm) containing three layers of solvents: (i, top) a ACN solution of $Cu(NO_3)_2{\cdot}3H_2O$ (1.4 μmol, 1.0 mL), (ii, center) a layer of DCM:MeOH:ACN (0.5:0.5:1 v/v/v, 1.0 mL), and (iii, bottom) a DCM:MeOH (1:1, v/v) solution of complex **1** (0.7 μmol, 1.0 mL). The light brown product was collected after 20 days at room temperature by centrifugation and washed with ACN and ethanol.

Formation of SolCON-B. A Wilmad® screw-cap NMR tube (⌀ = 5 mm and length = 17.8 cm) containing three layers of solvents: (i, top) an ACN solution of complex **1** (0.7 μmol, 1.0 mL), (ii, center) a layer of DMF: ACN (1:1 v/v, 1.0 mL), and (iii, bottom) a DMF solution of $Cu(NO_3)_2{\cdot}3H_2O$ (1.4 μmol, 1.0 mL). The light brown product was collected after 20 days at room temperature by centrifugation and washed with ACN and ethanol.

Formation of SurCON. The **SurCON** was formed by using an automated spin coater equipped with three syringes containing: (i) $Cu(NO_3)_2 \cdot 3H_2O$ (4.0 mM) in ACN, (ii) complex **1** (0.6 mM) in DCM/MeOH (1:1 v/v), and (iii) acetone for washing. The solution of $Cu(NO_3)_2 \cdot 3H_2O$ was drop casted (0.7-0.8 mL) onto FTO/glass (2 cm × 2 cm), subsequently, the substrate was spun at 500 rpm (acceleration 250) for 10 s and then at 1000 rpm (acceleration 500) for 30 s. Next, a solution of complex **1** was drop casted (0.7-0.8 mL) after 80 s on the substrate, which was spun as above. Then, acetone was drop casted after 80 s and spun for 40 s at 1000 rpm (500 acceleration). The $Cu(NO_3)_2$/**1** deposition steps is referred to as a deposition cycle. For the formation of the **SurCON** 18 deposition cycles were applied. The initial metal-salt deposition step on the metal-oxide surface is essential; spin coating first complex **1** did not afford a stable film.[S4]

Scanning electron microscopy (SEM) of SolCON. Samples were prepared by placing a drop of the reaction mixture on a silicon substrate and the solvent was allowed to evaporate. SEM measurements were performed using HRSEM ULTRA-55 ZEISS and HRSEM SUPRA-55 VP ZEISS instruments at an EHT voltage of 1.5 kV. Images were collected in secondary and backscattered electron modes by using Everhart-Thornley and energy selective backscattered detectors, respectively. The SEM-energy-dispersive X-ray spectroscopy (EDS) mapping was performed by using EDS Bruker XFlash/60 mm at accelerating voltages of 4 kV and 10-12 kV. The aperture size was 30 microns. The surface of sample was coated with 5 nm-thick carbon layer.

UV/Vis Spectroscopy of SurCON. A Cary 100 spectrophotometer was used to record UV/Vis absorbance spectra using Cary Win UV-Scan application program, version 3.00 (182) by Varian (200-800 nm). The transmittance was measured using the Cary Win UV-Kinetics application program, version 3.00 (182) by Varian. Bare substrates were used to compensate for the background absorption.

Transmission Electron Microscopy. Samples of **SurCON** on FTO/glass were prepared by sectioning in a Helios Focused Ion Beam Microscope (Thermo Fisher Scientific Microscopy Solutions,TFS, Hillsborough, USA). The sample was first coated with a 300-nm-thick layer of carbon, followed by coating a 350-400 nm-thick layer of platinum using electron-beam-assisted deposition. This process was followed by anion-beam-assisted deposition of a 200-250-nm-thick layer of platinum. The platinum coating protects the **SurCON** from ion-beam damage. A TFS

Tecnai F20 Twin (200 kV, FEG) instrument was used to collect energy dispersive X-ray spectrum images (EDS) on an EDAX Phoenix Si(Li) detector. High-angle annular dark field images were collected on a Fischione Model 3000 detector. For the analysis of **SolCON**, a suspension (10 μL) of as-synthesized crystals was dispersed on lacey carbon support on molybdenum grids for TEM analysis. Nanobeam electron diffraction (NBED) data were obtained in a double aberration-corrected TFS Themis-Z microscope, equipped with a high-brightness FEG at an acceleration voltage of 200 kV. For the 4D-STEM recording, the sample was kept at liquid N_2 temperature in a Gatan 914 cryo-holder (Gatan Inc., Pleasanton/CA, USA) to avoid radiation damage to the crystals. The EMPAD (electron microscope pixel array detector) allowed rapid data collection of the entire unsaturated diffraction pattern with a pixel dwell time of 1 μs for each pattern. An electron probe with a convergence angle of 0.2 mrad was adjusted in STEM microprobe mode and further defocussed by typically 5-10 μm to reduce probe size and electron flux. A primary beam current of less than 4 pA was used. Typically, diffraction patterns were acquired over a raster of 128 × 128 pixels. The total exposure in the 4D-STEM experiments was approximately 1 e/Å^2.

X-ray Photoelectron Spectroscopy (XPS) of SurCON. FTO/glass (1.0 cm × 1.0 cm) was used for XPS measurements using a Kratos AXIS ULTRA system, having a monochromatic Al Kα X-ray source (*hv* = 1486.6 eV) at 75 W and detection pass energies ranging between 20 and 80 eV. Curve-fitting analysis was based on Shirley or linear background subtraction and the application of Gaussian-Lorentzian line shapes.

Electrochemical characterization of SurCON. The electrochemical measurements were carried out using a CHI660A or CHI760E electrochemical workstation. The modified FTO/glass served as the working electrode, Ag/Ag^+ was used as the reference electrode, and a Pt wire as the counter electrode. Tetrabutylammonium hexafluorophosphate ($TBAPF_6$), in ACN (0.1 M) was used as the supporting electrolyte.

X-ray crystallography. A single crystal of **SolCON-B** suitable for X-ray diffraction was coated with Paratone oil (Hampton Research, CA, USA), mounted on a MiTeGen loop and flash frozen in liquid nitrogen. Diffraction data were recorded on Rigaku Synergy system equipped with a Dectris Pilatus 300K Cd-Te detector. Data were measured with MoK_{α} radiation at 100(2) K. The data were collected and processed with Rigaku OD CrysAlisPro 1.171.40.60a (2019). The structure was determined by direct methods using SHELXT-2018 with SHELXL-2013 and SHELXL-2016/4.[S5-S7] The crystal data have been deposited with the CSD (**Table S1**).

Table S1. Crystal data and structure refinement of SolCON-B.

Formula	$C_{288}H_{216}Cu_{12}N_{60}O_{48}$
CCDC	2095187
Formula weight	6047.68 (g/mol)
Temperature	100 K
Wavelength	0.71073 Å
Crystal system	Monoclinic
Space group	*C*2/*m*
Unit cell dimensions	a = 25.9724(9) Å α = 90°
	b = 20.8477(10) Å β = 93.339°
	c = 20.9552(8) Å γ=90°
Volume	11327.2(8) $Å^3$
Z	1
Density (calc)	0.887 mg/m^{-3}
Absorption coefficient	0.603 mm^{-1}
F (000)	3096
Crystal size	0.120 mm × 0.120 mm × 0.080 mm
Theta range	1.59° to 30.23°
Index range	$-36\leq h\leq 29$, $-27\leq k\leq 29$, $-28\leq l\leq 29$
Reflections collected	73402
R_{int}	0.1245
Completeness (%)	99.8
Data/restraints/parameter	14847/0/468

Goodness-of-fit on F^2	0.943
Final R [I>2σ(I)]	R_1=0.0871, wR_2=0.2262
R (all data)	R_1=0.1321, wR_2=0.2403
Largest diff peak and hole	2.559 e•Å^{-3} and -0.569 e•Å^{-3}

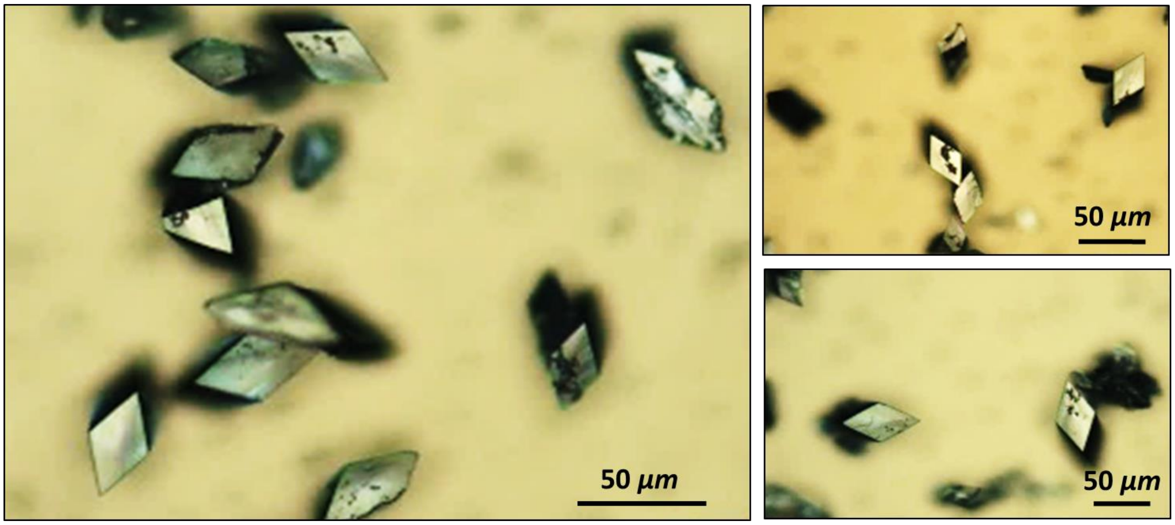

Figure S1. Representative optical microscopy images of **SolCON-B**.

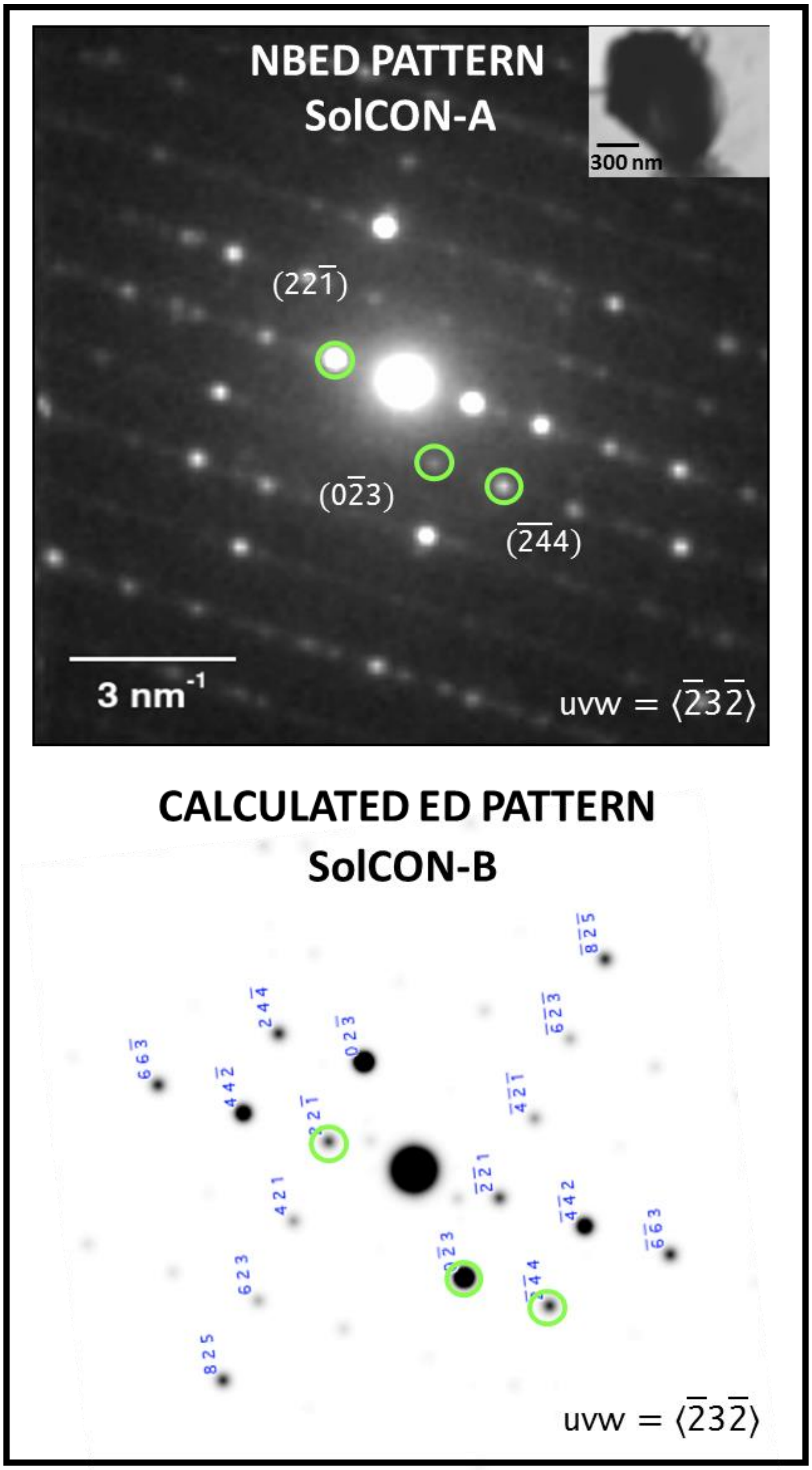

Figure S2. (Top) Experimental nanobeam electron diffraction (NBED) pattern of **SolCON-A**. (Bottom) Simulated electron diffraction (ED) from the single-crystal data of **SolCON-B**. Circles indicate matching diffractions.

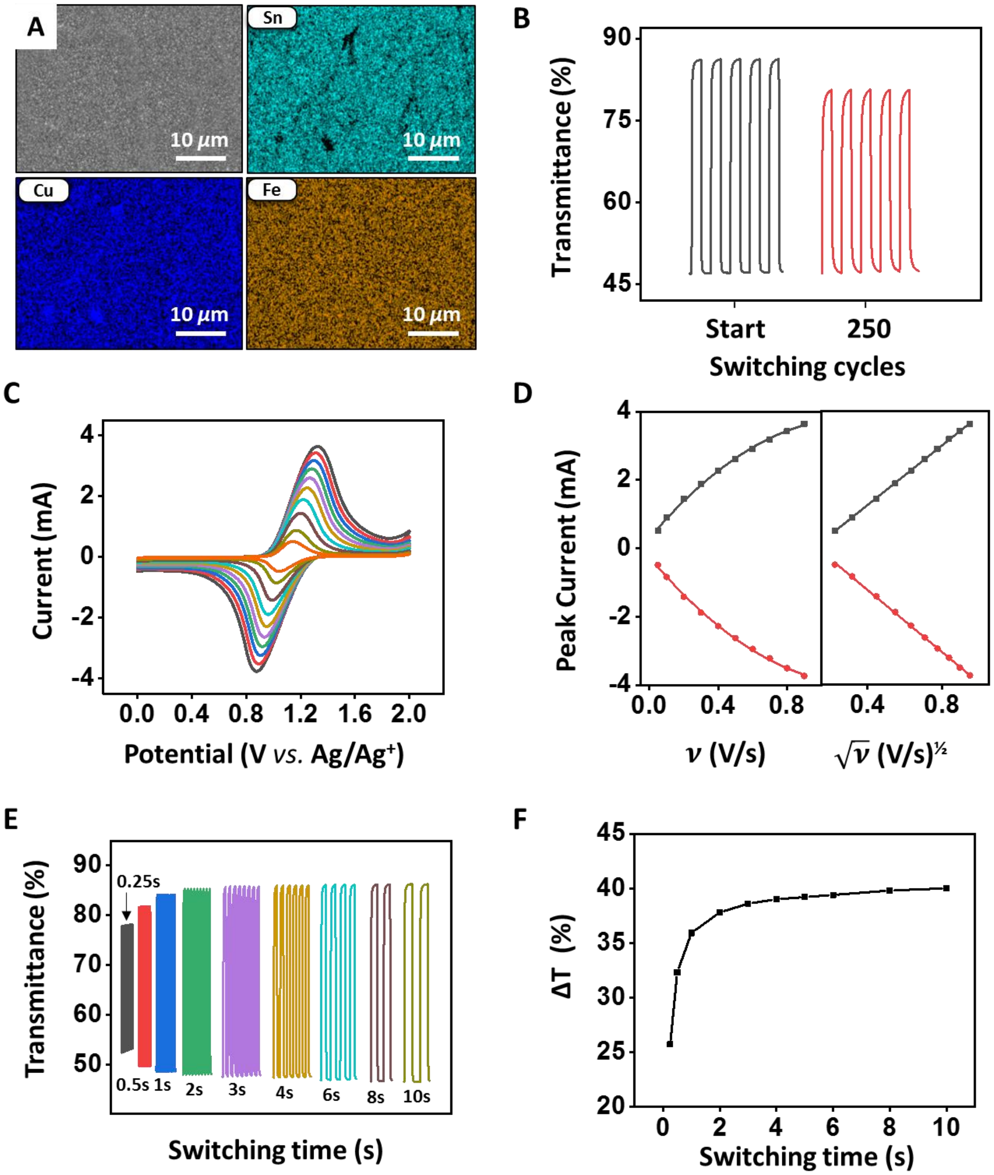

Figure S3. Spectroelectrochemical (SEC) performance of the **SurCON** on FTO/glass (2 cm × 2 cm) in a 0.1 M $TBAPF_6$/ACN electrolyte solution using Pt wire and Ag wire, as counter and reference electrodes, respectively. (A) EDS-SEM images. (B) SEC using double potential steps: 0.4-1.8 V (λ_{max} = 596 nm). (C) Cyclic voltammograms (CVs) with scan rates of 0.05-0.9 V/s. (D) Exponential and linear correlations between the peak currents (I) and scan rates (v) (left), and I and $v^{1/2}$ (right) respectively, during oxidation (top) and reduction (bottom) (R^2 > 0.99 for all fits). (E) SEC measurements using double potential steps: 0.4 V to 1.8 V at different switching times (F). Dependence of the contrast ratio (ΔT) on the switching time.

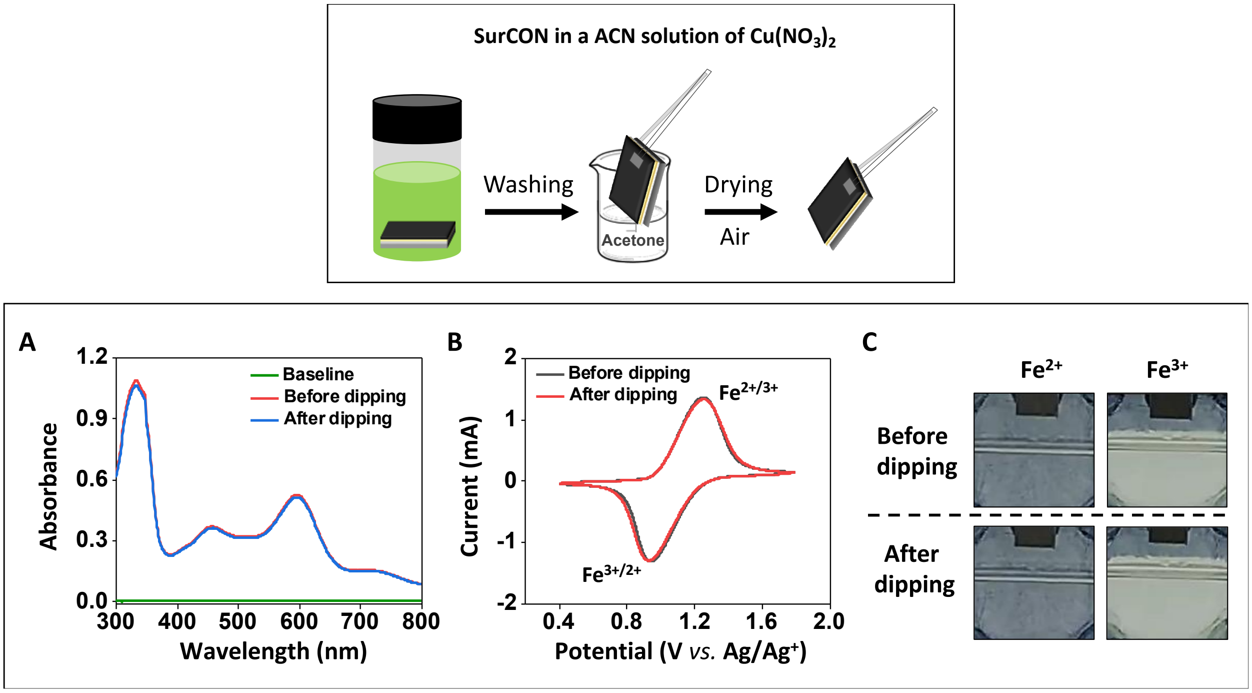

Figure S4. The surface and electrochemical characterization of **SurCON** before and after immersion in a 4.0 mM solution of $Cu(NO_3)_2·3H_2O$ in ACN for 3 days. (A) UV/Vis absorption spectra, (B) Cyclic voltammograms (CVs) recorded at a scan rate of 100 mV/s, (C) Photographs of the colored and bleached states using an electrolyte solution of 0.1 M $TBAPF_6$ in ACN. Reduced state (Fe^{2+}, 0.4 V) and oxidized state (Fe^{3+}, 1.8 V).

Chart 1 **Chart 2**

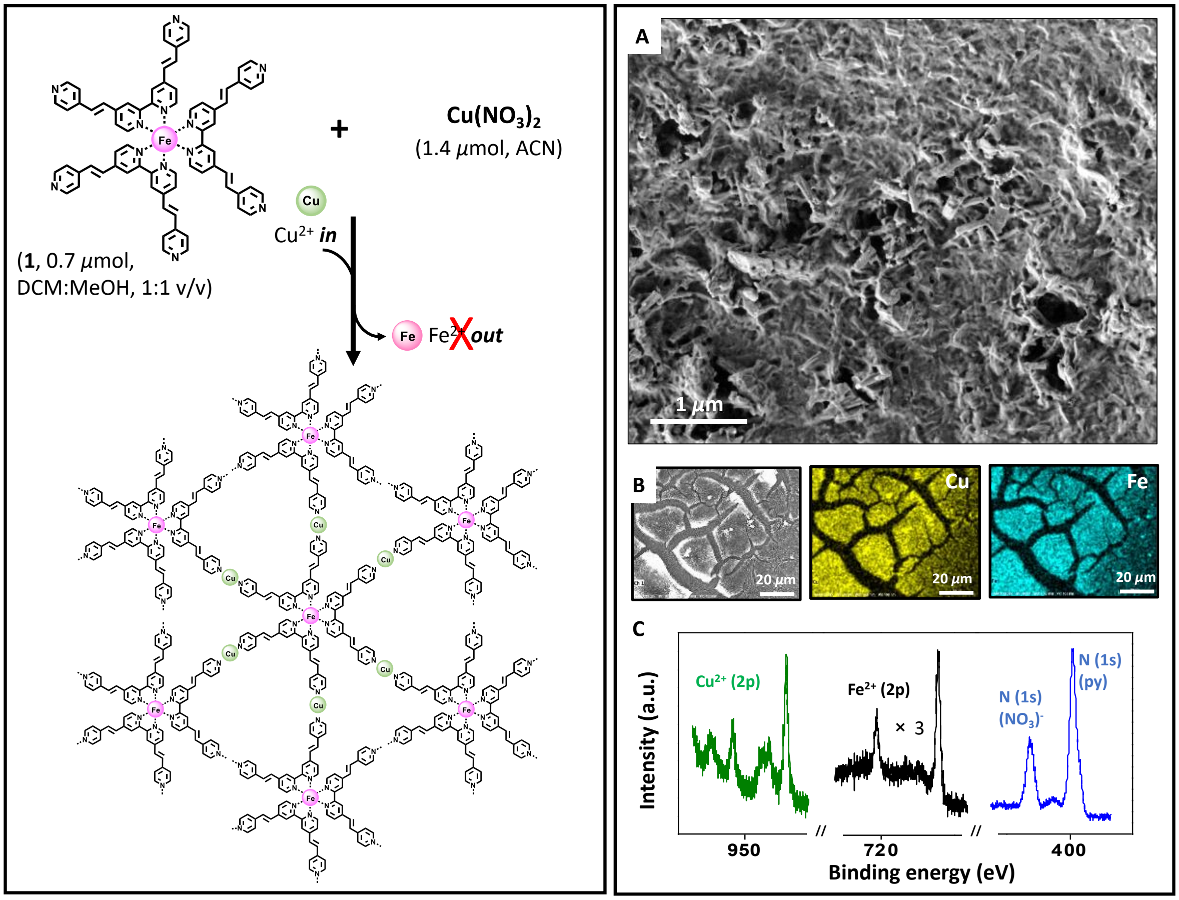

Figure S5. A solution of complex **1** (0.7 μmol, DCM:MeOH, 1:1 v/v, 1.0 mL) and $Cu(NO_3)_2·3H_2O$ (1.4 μmol, ACN, 1.0 mL) were mixed at room temperature. **Chart 1**: Reaction of $Cu(NO_3)_2$ and iron complex **1**, and proposed structure of the formed network. **Chart 2**: (A) Scanning electron microscopy (SEM) image. (B) SEM and EDS-SEM images. (C) X-ray photoelectron spectroscopy (XPS) spectra. The observed ratio of Cu/Fe is ~1.5 and N/Fe is ~10.5.

Chart 1 **Chart 2**

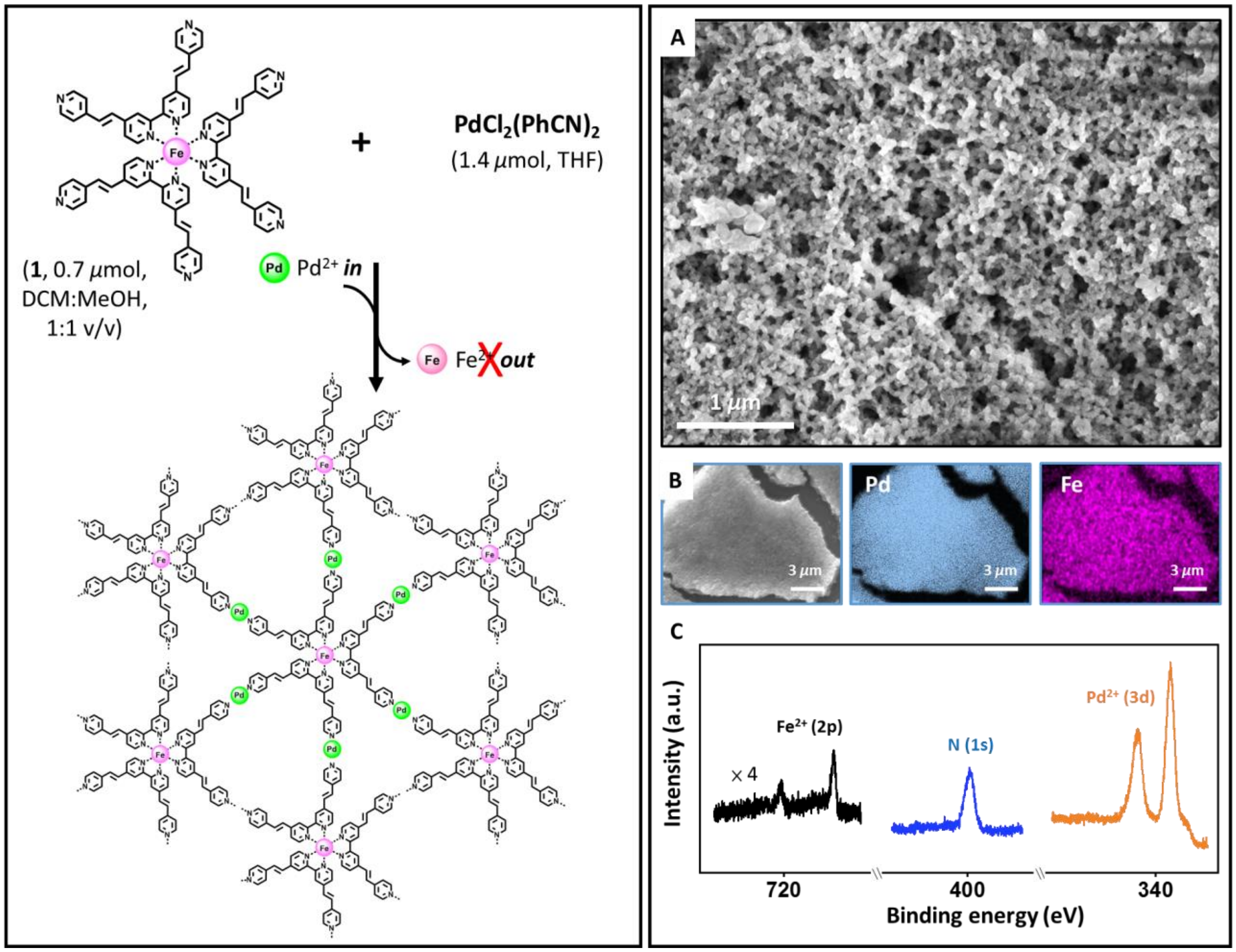

Figure S6. A NMR tube containing three layers of solvents with complex **1** and $PdCl_2(PhCN)_2$ was kept for more than 20 days at room temperature: (top) a THF solution of $PdCl_2(PhCN)_2$ (1.4 μmol, 1.0 mL), (center) a layer of DCM:MeOH:THF (0.5:0.5:1 v/v, 1.0 mL), and (bottom) a DCM/MeOH (1:1, v/v) solution of complex **1** (0.7 μmol, 1.0 mL). **Chart 1**: Reaction of $PdCl_2(PhCN)_2$ and iron complex **1**, and proposed structure of the formed network. For a related study, see reference S8. **Chart 2**: (A) Scanning electron microscopy (SEM) image. (B) SEM and EDS-SEM images (C) X-ray photoelectron spectroscopy (XPS) spectra. The observed ratio of Pd/Fe is ~3.9 and N/Fe is ~12.1.

References

S1. N. Malik, N. Elool Dov, G. de Ruiter, M. Lahav, M. E. van der Boom, *ACS Appl. Mater. Interfaces* **2019**, *11*, 22858–22868.

S2. http://www.steinlabs.com/technical-principles.html

S3. https://www.ossila.com/pages/sheet-resistance-theory

S4. Elool Dov, N.; Shankar, S.; Cohen, D.; Bendikov, T.; Rechav, K.; Shimon, L. J. W.; Lahav, M.; van der Boom, M. E. *J. Am. Chem. Soc.* **2017**, **139**, *33*, 11471–11481.

S5 Sheldrick, G.M. "SHELXT - integrated space-group and crystal-structure determination", *Acta Cryst.* **2015**, A71, 3

S6 G. M. Sheldrick, SHELXT-2013, Program for the Solution of Crystal Structures; University of Göttingen, Göttingen, Germany **2013**.

S7 G. M. Sheldrick, *Acta Cryst*. **2015**, *C71*, 3–8.

S8 I. Mukkatt, P. M. Anjana, A. Nirmala, R. B. Rakhi, S. Shankar, A. Ajayaghosh, Mater. *Today Chem.* **2020**, *16*, 100260.
